# Video Documentation in Thyroidectomy and an Evaluation of Operative Notes

**DOI:** 10.7759/cureus.64446

**Published:** 2024-07-13

**Authors:** Mazin Merdad, Hoda Alsayid, Shouq Alsharif, Almoaidbellah Rammal, Nada J Farsi, Hani Z Marzouki

**Affiliations:** 1 Otolaryngology - Head and Neck Surgery, Faculty of Medicine, King Abdulaziz University, Jeddah, SAU; 2 Medicine, King Fahad Armed Forces Hospital, Jeddah, SAU; 3 Dental Public Health, Faculty of Dentistry, King Abdulaziz University, Jeddah, SAU

**Keywords:** general otolaryngology, otolaryngology-head & neck surgeons, operative notes, video documentation, thyroidectomy

## Abstract

Introduction

Accurate and detailed documentation of surgical operation notes is crucial for post-operative care, research and academic purposes, and medico-legal clarity. Several studies have shown their defiency and inaccuracy sometimes, and some methods have been proposed to make them more objective. This study aimed to evaluate the completeness of thyroidectomy operative notes in a tertiary center and to assess the adequacy of video documentation by comparing it to the corresponding operative notes.

Methods

A retrospective review of thyroidectomy operative notes from 2010 to 2020 at King Abdulaziz University Hospital, Jeddah, Saudi Arabia, was performed to ensure completeness. Subsequently, 15 thyroidectomies were video recorded, and their notes were compared to the corresponding written operative notes. The completeness score was calculated based on an item list that included items that had to be included in an operative note. An independent samples t-test was used to compare the completeness score means between the two groups. One-way analysis of variance was used to compare the completeness score means between two or more groups.

Result

A total of 385 thyroidectomy-operative notes were retrospectively reviewed. The completeness scores ranged between 6% and 89% for the various items that had to be documented, with a mean of 54.47%. The mean score of the video-documented operative record was 83.86%±12.84%, which was significantly higher than the corresponding written operative notes (47.53%±18.06%) (p <0.001).

Conclusion

Video documentation showed significant improvement compared to the corresponding written and retrospective operative notes. Video recording can also be a valuable tool when teaching anatomy and surgical skills and conducting research.

## Introduction

Documentation is defined as any material that describes an event. Medical documentation initially started as an effort toward education. Some early examples include an Egyptian case report from 1600 BC, Hippocrates from 460 BC, and Al-Razi from 860 CE. Medical records have since evolved significantly and have become a part of official hospital records by 1898 [1].

Medical records are fundamental for clinical care and audit of surgical services. Operative notes remain one of the vital medical records. They must be prepared immediately after surgery and contain descriptions of surgical procedures, techniques, findings, reasons for making specific decisions, instruments, the material used in surgery, and any events encountered during the procedure. Proper documentation of a patient’s history and operative procedures is essential for all healthcare providers [2,3]. This is the primary method of communication between different medical services [4,5]. It also ensures quality care and follow-up [2,4]. Above all, they are considered a key medicolegal document [2-7]. Furthermore, surgical quality, which can be determined from operative notes, has a significant prognostic value [8,9]. Currently, documentation of technical information in any surgical procedure depends on operative reports, which are highly subjective, mutable, and lack key details [2,4,9,10]. Traditionally, operative notes were the only method for surgical documentation; however, with the rapid development of multimedia technology in healthcare systems, it has rapidly changed.

Many methods have been proposed and studied to make operative reporting more objective and reliable [7,8,10-17]. While video recording has become increasingly feasible and accurate, most of the surgeries studied thus far are endoscopic procedures [14-17]. However, it is essential to assess video documentation during open surgeries. Moreover, video recording can be used in education to teach anatomy and surgical skills, as an assessment tool for evaluating surgical skills, and for research purposes [18]. Nevertheless, as with any change, video recording of surgical procedures has been met with skepticism [13]. However, as O’Mahoney et al. believe, this could be the next driver for surgical quality [13].

Thyroid cancer is one of the five most common cancers worldwide [19]. It was also the third most common cancer in 2020 in Saudi Arabia with 2,833 new cases (10.2%), colorectal cancer (14.4%), and breast cancer (14.2%), as reported by the International Agency for Research on Cancer - World Health Organization [19]. It is the second most common cancer in women (14.2%) after breast cancer (29%) [19]. This is reflected by the high number of thyroidectomies performed in the Kingdom of Saudi Arabia, which is a relatively common procedure for thyroid cancer. In the National Guard Hospital in Jeddah, 456 thyroidectomies were performed between 2008 and 2017 [20]. In Aseer Central Hospital, 150 total thyroidectomy surgeries were performed between 2000 and 2019 [21]. Between 2011 and 2018, 320 studies were conducted at King Fahad Central Hospital in Jazan [22].

With the increasingly litigious nature of the medical practice, accurate documentation is critical, and errors of documentation are known to occur in all medical specialties with a possible range of clinical implications and medico-legal consequences. Therefore, this study aimed to evaluate the completeness of thyroidectomy operative notes at King Abdulaziz University Hospital, Saudi Arabia, between 2010 and 2020. In addition, the adequacy of the video documentation was assessed by comparing it with the corresponding operative notes. Thyroidectomy is one of the most common operations in the head and neck region and was the surgery of choice in this study. The findings of this study are of significant importance in improving the clinical practice for surgeons worldwide.

## Materials and methods

Study design

We conducted a retrospective review of thyroidectomy surgeries performed at a single tertiary center to assess the completeness of thyroidectomy operative notes. Ethical approval to conduct this study was obtained from the Biomedical Ethics Research Committee at King Abdulaziz University, Saudi Arabia (approval no: 604-20).

Data collection

Between 2010 and 2020, data were collected from King Abdulaziz University Hospital, Saudi Arabia, for patients who underwent surgeries booked under one of these codes (International Classification of Diseases Tenth Revision (ICD-10), Sixth Edition) (Table 1) [23].

**Table 1:** ICD-10 codes with the corresponding surgical procedures International Classification of Diseases Tenth Revision (ICD-10), Sixth Edition

Code	Surgical procedure
30296-00	Total thyroidectomy
30308-00	Subtotal thyroidectomy, bilateral
30309-00	Subtotal thyroidectomy for thyrotoxicosis
30297-00	Total thyroidectomy, following previous thyroid surgery
30297-01	Subtotal thyroidectomy, following previous thyroid surgery
30310-00	Subtotal thyroidectomy, unilateral
90046-00	Subtotal thyroidectomy, substernal
90046-01	Total thyroidectomy, substernal
30075-03	Biopsy of the thyroid gland
30306-00	Total thyroid lobectomy, unilateral
90041-00	Other procedures on the thyroid gland

For each surgery, demographic data on the patient, surgery name and date, team who performed the surgery, and diagnosis were collected. A general physician reviewed all the operative notes. To assess the completeness, a score was calculated for every operative note. The score was assigned based on a checklist of two studies [12,24]. These studies enlist the details that must be written in optimal thyroidectomy operative notes. A checklist based on these studies was created, reviewed, and approved by a head and neck surgeon.

The checklist has the following steps and findings: incision site and size, local anesthesia injection, raising the subplatysmal flap, strap muscle separation, side from which the surgeon started the procedure, thyroid gland or nodule description, invasion or extrathyroidal extension, the status of the recurrent laryngeal nerve (RLN) (both sides for total thyroidectomy cases and one in cases of hemithyroidectomy), use of nerve monitor, parathyroid glands present in each lobe (at least one), residual tissue left behind, sutures used for strap closure, sutures used for platysma and in skin closure, drain score (only in cases where the drain was documented in nurse sheet), dressing, and finally palpation or dissection of central lymph nodes (only in cases diagnosed with thyroid cancer).

The completeness of the operative report score represents the number of documented steps compared to the total number of steps on the checklist. The training level of the doctor who documented the operative notes and the duration between the surgery date and operative note dictation data were also collected.

Video recording data

For two consecutive months, all thyroidectomies performed were video- and audio-recorded using a portable camera and a microphone plugged into the main surgeon’s loup. In this study, we used an LED DayLite NanoCam HDi™ (Design for Vision, Inc., Bohemia, NY).

Fifteen surgeries were recorded, and the operative notes were written as usual for these cases by surgeons blinded to the study. Using the same checklist, operative note scores were assigned to the written operative notes for these 15 cases by the same general physician who evaluated these written operative notes for the retrospective part of this study. The same checklist was used to check the completeness of the video-recorded operative report. The operative record score was calculated by a third surgeon (a head and neck fellow), who reviewed all recorded videos and gave a completeness score for each case. The completeness of the operative reports was also analyzed. The written operative notes from the retrospective part of the study, documented videos, and the corresponding written operative reports from the 15 video-recorded cases were compared.

Statistical analyses

Frequencies and percentages were used to represent categorical variables. Means and standard deviations were used to illustrate continuous variables. The Shapiro-Wilk test was used to test the normality of the continuous variables (age and completeness score). The chi-square test was used to assess the associations between categorical variables. A two-sample t-test was used to compare the means between the two groups. One-way analysis of variance (ANOVA) was used to compare means between more than two groups. P-values less than or equal to 0.05 were considered statistically significant.

## Results

A total of 385 patients underwent thyroid surgery between January 2010 and December 2020 at King Abdulaziz University Hospital; of these, 313 (81.3%) were women. The mean age of the patients was 47 years (range: 11-86 years).

There was a clear trend of patients undergoing thyroidectomy becoming younger starting in 2015. The mean age, which was 55.5±15.1 for thyroidectomies performed in 2010-2014, was significantly higher than that in 2015-2020 (45.2±14.1) (p < 0.001). Approximately half of these cases, 213 (55.3%) were handled by the Otolaryngology-Head and Neck Surgery Teams (ORL-H & NS), while the remaining cases were handled by general surgery (GS) teams. The number of thyroidectomies performed by ORL-H & NS teams in 2015-2020 was significantly higher than that between 2010 and 2014: 63.1% vs. 21.8%, respectively (p <0.001). As demonstrated in Table 2, there was a statistically significant difference in preoperative vocal cord assessment between the ORL-H & NS and GS groups (p < 0.001).

**Table 2:** Number of cases with documented vocal cord assessment in Otorhinolaryngology-Head and Neck Surgery (ORL-H & NS) and General Surgery (GS) * The total number of participants does not add up to the total because of missing values.

Department	Preoperative vocal cords assessment		Total
	Undocumented N (%)	Documented, N (%)	
ORL-H & NS	99 (46.3%)	112 (66.7%)	211
GS	115 (53.7%)	56 (33.3%)	171
Total	214	168	382

Of most thyroidectomy procedures, 270 (70.1%) were total thyroidectomies, followed by hemithyroidectomies 100 (26%) and completion thyroidectomies 15 (3.9%). In 60 (15.6%) of cases, the reason for booking a surgery was different from the surgery performed. Regarding the diagnosis that led to thyroidectomy, 222 (57.7%) were performed for benign disease and 149 (38.7%) for papillary thyroid cancer. Meanwhile, 12 (3.1%) had follicular thyroid cancer, and two (0.5%) had medullary thyroid cancer.

Of the 385 surgeries, operative notes were not written for 33 (8.6%). These 33 surgeries were performed between 2010 and 2019 and were performed across both departments, ORL-H & NS and GS.

Table 3 illustrates the operative note checklist and percentages of documentation of each step for the remaining 352 surgeries. Operative note completeness scores were also calculated, as demonstrated in Table 4. The scores ranged from 6 to 89, with an overall mean of 54.47 (14.2). The completeness scores also varied by year.

**Table 3:** Operative note checklist and percentages of documentation of each step

Checklist	Documentation, N (%)	No Documentation, N (%)
Incision site	311 (88.4%)	41 (11.6%)
Incision size	111 (31.5%)	241 (68.5%)
Local anesthesia injected	78 (22.2%)	274 (77.8%)
Raising subplatysmal flap	281 (79.8%)	71 (20.2%)
Strap muscles separation	242 (68.8%)	110 (31.2%)
Starting with which side	303 (86.1%)	49 (13.9%)
Thyroid gland or nodule description	88 (25.0%)	264 (75.0%)
Invasion or extra thyroidal extension	30 (8.6%)	322 (91.4%)
Right recurrent laryngeal nerve	293 (83.3%)	59 (16.7%)
Left recurrent laryngeal nerve	289 (82.1%)	63 (17.9%)
Parathyroid glands at least in each lobe	215 (61.1%)	137 (38.9%)
Residual left behind	7 (2.0%)	345 (98.0%)
Palpation or dissection of central lymph nodes	145 (41.1%)	207 (58.9%)
Use of nerve monitor	95 (27.0%)	257 (73.0%)
Sutures used in strap closure	156 (44.3%)	196 (55.7%)
Sutures used in platysma closure	153 (43.6%)	199 (56.4%)
Sutures used in skin closure	166 (47.3%)	186 (52.7%)
Drain score	325 (92.3%)	27 (7.7%)
Dressing	339 (96.3%)	13 (3.7%)

**Table 4:** Operative note scores from 2010 to 2020 (n = 352)

Year	N (%)	Mean (SD)	Minimum	Maximum
2010	23 (6.5)	52.3 (18.8)	11	82
2011	11 (3.1)	50.4 (20.7)	6	74
2012	6 (1.7)	54.5 (8.0)	42	65
2013	5 (1.4)	44.0 (9.5)	29	53
2014	6 (1.7)	63.2 (16.0)	41	84
2015	25 (7.1)	45.2 (14.0)	28	76
2016	64 (18.2)	49.0 (11.1)	11	70
2017	44 (12.5)	51.7 (12.4)	21	76
2018	66 (18.8)	55.0 (13.5)	28	83
2019	60 (17.0)	60.9 (12.6)	33	83
2020	42 (11.9)	63.3 (14.9)	18	89

As illustrated in Table 5, the operative note scores for notes written by ORL-H & NS were higher than those written by GS (56.5 (12.9) vs. 51.9 (16.3); p < 0.001). The seniority of the physicians who wrote the operative notes was also assessed. Seniors scored the highest, followed by juniors, consultants, fellows, and specialists. Senior-level residents’ scores were significantly greater than fellows and specialists (p = 0.038).

**Table 5:** Operative note scores based on specialty and on seniority level (n = 352) *Two-sample t-test ^ One-way analysis of variance (ANOVA) and multiple comparisons indicated that senior-level residents’ scores were statistically significantly greater than those of fellows and specialists. The total number of participants does not add up to the total because of missing values.

Variable	N (%)	Mean (SD)	P-value
Specialty			
ORL-H & NS	201 (57.1)	56.5 (12.9)	<0.001*
GS	150 (42.6)	51.9 (16.3)	
Seniority			
Junior (PGY 1–3)	106 (30.1)	54.0 (14.3)	0.038^
Senior (PGY 4–5)	201 (57.1)	56.0 (14.4)	
Fellow/specialist	26 (7.4)	47.9 (16.0)	
Consultant	15 (4.3)	50.0 (14.6)	

The majority of operative notes were written on the same day as the surgery. However, in 28 (8%) of the cases, the duration between the surgery date and the operative note date ranged from 1 to 678 days. There was no statistically significant difference when the operative note score was compared for the diagnosis or duration between the surgery and operative notes. Moreover, documentation of nerve monitor usage increased in the period 2015-2020, compared to 2010-2014, (p < 0.001). RLN was documented in 291 (82.7%) of the cases.

Of the 15 surgeries that were video-recorded, 14 (93.3%) were total thyroidectomies, and only one (6.7%) was hemithyroidectomy. Nine cases were papillary thyroid cancer (60%), and six were benign (40%).

As shown in Table 6, among the 15 surgeries, the mean of the written operative note score was 47.5±18.1, which was significantly less than the corresponding video operative record (83.9±12.8) (p < 0.001), with a mean difference of 36.4 and 95% confidence interval (CI) of (24.4-48.2). The operative note completeness was the highest in the video-recorded documentation group compared with the written operative notes of the same 15 cases and compared with the retrospective group.

**Table 6:** Summary of the mean operative note scores for the written notes of the retrospective group (2010-2020), the written operative notes of the study group, and the video-recorded documentation of the study group P-values ≤ 0.05 are considered statistically significant.

	Group A retrospective group	Group B study group (written operative note)	Group C study group (video-recorded documentation)	Group A*B P-value	Group B*C P-value	Group A*C P-value
Mean of operative note completeness score	54.47 (14.2)	47.53 (18.1)	83.86 (12.8)	0.116	<0.001	<0.001

## Discussion

Medical records are fundamental to medical care. These medical records include admission notes, daily progress notes, laboratory test results, radiological reports, operative reports, informed consent documents, and discharge summaries. Proper and complete documentation in all these records is essential for good patient care. This study assessed the completeness of thyroidectomy open-surgery operative notes in a tertiary center between 2010 and 2020. We also assessed the completeness of the video documentation by comparing it with the corresponding operative notes.

Operative documentation types

Narrative and synoptic reports are the two main types of operative report documentation. A narrative operative note is a traditional document written in a descriptive, story-like manner. Such documentation is highly variable and lacks standardization and important details. Synoptic reports, which have been introduced recently, are becoming increasingly common in clinical practice. These notes have formats used with specific data elements to reduce variability. These templates have been shown to improve the completeness of the reports [10,11,25-28]. Two systematic reviews demonstrated that synoptic reports were significantly more complete, reliable, and faster and may provide a cost-benefit over narrative reports [2,29].

Importance video recording

Operative video recording proved to be useful in many aspects. Reviewing the video for root-cause analysis [13], tracking errors [16], reflecting on the quality of surgery [7], morbidity and mortality meetings [30], and improving communication between surgeons and oncologists [14,15].

An example to improve our understanding of the development of complications was reported in dacrycystorhinostomy. In this retrospective review, endoscopic dacryocystorhinostomy surgeries were video recorded. Later, using these videos, surgical steps were analyzed, which revealed the possible reasons for procedure failure [31]. This has extreme importance as up to 500,000 deaths each year are due to perioperative complications that are said to be potentially preventable [16]. Near-miss events are rarely documented in written operative notes [7,30].

This is why routine video recording for all cases has been proposed [32] to allow accident analysis, which will eventually improve surgical care and decrease complications. Using video recording routinely for documentation was emphasized again in 2019 to identify causes for poor surgical outcomes, which cannot be done using traditional written operative notes [7]. Video recording was also highlighted as a way to reduce variability in surgical outcomes between local and national levels [13].

The Hawthorne effect (which is a modification in an individual's behavior due to awareness of being observed) may also play a role in improving surgical outcomes in video-recorded cases [7]. An example of this effect was evident in colonoscopy, where the quality of mucosal inspection improved by 31% in video-recorded cases [33].

Apramian et al. reached this conclusion, "What happens in the operating room is not necessarily reflected in detail in the operative note", which reflects the unreliability of written operative notes and the possibility of being misinterpreted by patients and lawyers [34]. The medicolegal aspect of this new method of documentation is not clear yet as policies are different between countries [7].

Regarding the concern of using this video documentation in malpractice suits, this, in fact, should be beneficial [16] and will give legal support to surgeons. It can actually be used for defense in medicolegal suits as it is an objective document [7]. However, surely, hospitals have the option to use these operative records for quality improvement purposes only and not as legal documents. This can be done after declaring that prior to video recording the procedure [30]. Like any other patient data, operative video records should be stored and retrieved as needed. Treating it as radiological images has been suggested [30]. Hospitals have to ensure the safe storage of these video documents like any other electronic data. It has to be secured using cybersecurity, digital security methods, or even physical security if hard devices are used for storage [7,35].

Video recording

With the rapid development of multimedia technology in healthcare systems, several methods have been proposed to make operative notes more objective and reliable. Video recording has been increasingly shown to be feasible and claimed to facilitate accurate documentation [17]; however, all these examining the feasibility were endoscopic surgeries [14-17]. An additional benefit of video-recording surgeries is that the recording of procedures positively impacts surgeons’ behaviors [33,36,37].

A study published in 2015 [15] included 16 laparoscopic colon surgeries. The study concluded that video documentation of the key surgical steps could improve patient care due to better communication with other physicians regarding anatomical changes and follow-up, as well as improvement in surgical training. Another study on colorectal surgery [14] showed some resemblance to our study. They compared the adequacy of video recording during surgeries with operative notes by using completed step documentation as a scoring system. They found that video-recorded surgeries and those combining video recording with operative notes recorded significantly more information than operative notes. Similarly, in our study, the group with video-recorded surgeries had a higher completeness score (83.86%) than that of surgeries documented by operative notes only (54.47%) [14]. Video recordings from both studies had a higher score, which implemented a more complete detailed document compared to written operative notes. Although our study was done in an open surgical procedure, our results were similar to the endoscopic study.

In 2020, JAMA Surgery published a study that included 79 laparoscopic cholecystectomy cases that were both video- and audio-recorded [17]. There was a significant improvement in the adequacy of documentation when video recording was used. The number of steps documented in the operative notes was 78%, compared with 92.3% in the video recordings. This was similar to our study results, where the percentage of documented steps in the operative notes was 47.5%, compared to the corresponding video recording for the same cases (83.86%). Again, although our study was done in an open surgical procedure, it showed similar findings as those of endoscopic ones. Concluding that video documentation whether it is for open or endoscopic surgeries has a significantly higher score than corresponding written operative notes. This encourages the use of video recording for surgical documentation and gaining all the benefits of video recording that were previously discussed.

Retrospective data

Maintaining complete surgical documentation is the responsibility of all surgeons. As with most healthcare facilities worldwide, our center does not implement specific teaching for proper operative note-writing during residency. In a survey conducted on obstetrics and gynecology residency programs in the USA, only 23% of respondents had formal training on operative dictations [38]. A national survey of surgical program directors in the USA revealed that only 17% of programs provided operative report documentation instructions [39]. Similar to any skill, operative note-writing requires formal teaching, which improves outcomes, as has been reported earlier [38,40].

A review of the existing literature on operative notes suggested that the documentation of operative notes is poor in different countries and in different surgical specialties. A study conducted in 2008 audited 190 operative notes for completeness and found that 51.6% of notes were incomplete [41]. Another study evaluating laparoscopic cholecystectomy operative notes in nine hospitals revealed that only 52-69% of the items in the checklist were documented [4]. A systematic review of the completeness of synoptic operative reports compared with narrative operative reports in surgery showed that synoptic operative reports were more complete than operative reports. Furthermore, the completeness of narrative reports for colon cancer and breast cancer surgeries were 31.7-45.9% and 45-66%, respectively [2]. In our study, the overall completeness of operative notes was 54.47%, which improved significantly with video documentation (83.86%).

The practice of documenting operative notes among trainees has been studied previously. In a study conducted in Washington, USA, junior resident operative notes were found to be more underdocumented than those of senior residents and staff [42]. A study from 2004 showed results similar to ours, wherein the dictation by residents was more complete than the consultants [11]. A blinded evaluation of the accuracy of 50 operative notes showed that operative reports dictated by senior residents were incomplete and inaccurate compared to those of attending surgeons [43]. A review of the operative notes of 24 laparoscopic cholecystectomy cases found no difference in the completeness of reporting by residents of different levels [44].

In another study, 158 operative notes of rectal cancer surgeries were assessed for completeness [45], and the overall adherence to the checklist items was found to be 55%. The overall adherence was higher among the attending staff members (67%) than among the residents (51%). Senior residents also showed higher compliance with the checklist (55%) than junior residents (44%). As against our study, senior residents had the most completed operative notes (56%), followed by junior residents (53.9%), consultants (50%), fellows, and specialists (47.9%).

We should also be mindful that, in our study, the training level of the doctor who documented the notes was captured from our electronic database. At our center, the doctor’s name is assigned to a record based on the user account in which the note was recorded since every doctor has their own username and password. In the retrospective evaluation part of our study, it was not possible to determine whether any doctor used an account belonging to a different doctor.

In our study, most of the operative notes were written on the same day of surgery; however, in 8% of the cases, the duration between the surgery date and the operative note documentation date ranged between one and 678 days. Although there was no significant difference in completeness when operative notes were written before or 24 hours after surgery, the accuracy of operative notes written 678 days after surgery was questionable. According to Pouliquen [46], any operative report written within a week of surgery is considered an operative memory report; two weeks later, it is considered an operative evaluation report; and beyond two weeks, it is considered an operative imagination report [46]. Another study showed that dictation was more deficient when delayed beyond 24 hours [43]. A study examining the timing of the day of operative note-writing did not reveal a significant difference in terms of completeness for 24 laparoscopic cholecystectomy surgeries [44].

In 2013, a 90-minute teaching session on basic and common mistakes in coding and billing was provided by an Otolaryngology-Head and Neck Surgery Department, and coding skills have been found to improve after a single surgery session [47]. It is essential to train healthcare staff in coding skills to improve coding accuracy and thereby improve patient prognosis.

Importance of this study

Although histopathology is the primary determinant for a treatment plan, intraoperative details and anatomical findings are important to optimize patient management, influence risk stratification, and develop follow-up plans since postoperative treatment can change based on the surgeon’s operative report. A communication gap between pathologists and other physicians was evident, as reported in a study by Powsner et al. [48]. In thyroidectomy cases, intraoperative findings could help determine the need for postoperative radioactive iodine treatment, reoperation, and factors to be considered during reoperation. Two intraoperative findings (local invasion and incomplete resection) were used to calculate prognostic factors [2,8].

In a study conducted by Chambers et al. in Alberta, Canada [8], among 271 thyroidectomies, the percentages of total thyroidectomy, thyroid lobectomy, and completion thyroid lobectomy were 73%, 16%, and 10%, respectively. This is similar to our study results, where the percentages were 70%, 26%, and 4%, respectively. Among the 271 thyroidectomies studies by Chambers et al. [8], the presence or absence of tumor invasion for a given case was only noted in 27% of the operative notes cases. In the retrospective part of our study, the presence or absence of tumor invasion was documented in 8.6% of the cases and 85.7% of the video-recorded cases. Another important element was the completeness of resection, which was documented in only 3% of the cases in the study by Chambers et al. [8]. Completeness of resection in our study was documented in 2% of retrospective data and 92.9% of video-documented notes. Finally, tumor size was documented in 29% of cases in the study by Chambers et al. [8]. In our study, thyroid gland or nodule description was reported in 25% and 100% of the cases in the retrospective evaluation and video-documented cases, respectively. Regarding the anatomical findings, the two most important findings were the RLN and parathyroid glands. RLN was documented in >95% of studies, 82% of retrospective studies, and 96% of video recordings. The parathyroid was documented in >83% of studies, 61% of retrospective studies, and 85.7% of video recordings (Table 7).

**Table 7:** Comparison between the study by Chambers et al. and two parts of our study Ref. [8]

	Chambers et al. [8], Canada	Retrospective data	Study group - Video notes
Tumor invasion	27%	8.6%	85.7%
Completeness of resection	3%	2%	92.9%
Tumor size	29%	25%	100%
RLN status	>95%	82%	96%
Parathyroid glands status	>83%	61%	85.7

Vocal cord assessment

In the same study by Chambers et al. [8], preoperative vocal cord assessment was performed in 72% of the patients who underwent thyroidectomy by an endocrine surgeon. Our study’s overall preoperative vocal cord assessment was poor, with only 43.8% of cases documented between 2010 and 2020. Furthermore, we found that only 8.2% of the cases were documented between 2010 and 2015 as against 52.3% during 2016-2020, showing a significant improvement in documentation over the years. Additionally, there was a significant difference in the preoperative vocal cord assessment between the ORL-H and NS teams compared to the GS team, in favor of the former.

Secondary outcomes

Video recording can be used to evaluate operative notes instead of or in combination with auditing. It can also be a valuable tool to teach both anatomical and surgical skills. Eighteen students who watched a narrated video recording of thyroidectomy using the same camera used in our study had significantly higher test scores than 19 students who studied only using a traditional textbook [18]. Video recording surgeries can also be used to evaluate surgical skills and self-training and can be used to assess the competency of residents in performing procedures.

Limitations and strengths of the study

Up to our knowledge, very few published articles have studied the benefits of operative video recording prospectively [14]. Compared to previous studies done in that field, which were mostly for endoscopic surgical procedures [14-17], in our study, we compared operative video documentation and corresponding written operative reports in an open surgical procedure (thyroidectomy). Studying the completeness of video documentation compared to written operative notes in this paper will serve as the foundation and base for establishing video recording as a standard method of documentation in open surgical procedures, where all discussed benefits of video documentation can be implemented. Moreover, this video recording can be used in education for both anatomy and surgical skills. It can also be used as an assessment tool for evaluating someone’s surgical skills, providing feedback, and for research purposes [16,18,30].

The cost of video equipment and the management of video recordings remain the two key limitations of our study. Another limitation of our surgery is that only a few surgeries were video recorded. This was due to decreased operating room times due to the COVID-19 pandemic during this study. Furthermore, in our study, the video recording focused on the documentation of surgical details, and the video recording was initiated after the patient was prepped and draped. However, surgical operative notes are not only limited to technical surgical details and contain other important elements. Data such as patient identification, patient position, type of anesthesia, and name of the anesthetist, surgeons, and nurses are all important elements of operative notes, which were not documented in our video recordings. Our study recommends combining written operative notes and video recordings to document surgical procedures more accurately.

## Conclusions

In our study, a retrospective evaluation revealed poor documentation of operative notes, highlighting the need for routine professional training to improve the quality of operative notes. Video recording can be used as a complementary tool to document surgical details to address the deficiencies of written operative reports or can be used as a tool to evaluate operative notes as an alternative to audits. Video recording can be a valuable tool for teaching anatomy and surgical skills, as well as for conducting research.

This study hopes to serve as a foundation in establishing video recording as a standard method of documentation in open surgical procedures, where all discussed benefits of video documentation can be implemented.
